# Tom Frieden: strategic interventions for maximum health impact

**DOI:** 10.2471/BLT.18.030118

**Published:** 2018-01-01

## Abstract

Tom Frieden tells Fiona Fleck why countries that focus on a few carefully selected public health interventions to reduce noncommunicable diseases will save the most lives.

Q: How did you become interested in public health?

A: Hiking through the mountains with my father, he commented that I was interested in health and politics and that public health combined both. When I went to medical school I already planned to go to into public health. My father, an excellent physician, had a simple philosophy of life: you’ve got to help the people. I chose a career that would give me the privilege of helping as many people as I could.

Q: How did you come to take on the emergence of multidrug-resistant tuberculosis in New York State in the early 1990s?

A: Tuberculosis found me, rather than the other way round. It started with an epidemic in New York City (NYC). Most outbreaks are found by an alert clinician. Dr Karen Brudney called me to say that she had noticed an increase in multidrug-resistant tuberculosis. Many people with HIV were developing active tuberculosis, but all forms of tuberculosis were spreading because of poverty, homelessness and poor infection control in hospitals. The national and local tuberculosis control programmes had been dismantled. In NYC, we provided services including directly observed treatment by health-care workers to ensure patients were cured. We also improved infection control in hospitals, and implemented the World Health Organization’s (WHO) system of rigorous review of every patient diagnosed. Once we implemented these programmes, case rates fell rapidly.

Q: That led to work with the WHO tuberculosis programme in India. Was the NYC experience applicable in other countries?

A: Some things are globally applicable, such as WHO guidelines and the importance of what I call a technical package. You can’t do everything, but if you focus on a limited set of interventions that are feasible to implement, scalable, and high-impact, you can make the maximum difference. For example, DOTS (directly observed treatment, short-course) did that with five key things for tuberculosis control: political and financial commitment, accurate diagnosis, directly observed treatment, a regular drug supply, and standardized recording and reporting of results. The basic concepts of high quality diagnosis, high quality treatment, and accountability for every patient’s outcome are widely applicable for infectious and noncommunicable diseases.

“Politics and public health are the art of the possible: you figure out what can be done and how, and then do it.”

Q: Can you tell us about your work in New York City under Mayor Bloomberg?

A: Mayor Bloomberg demonstrated how important political leadership is for success in public health. When I brought issues to him, his key question was: “Are you certain this will save lives?” And if I could answer “Yes,” he replied: “Then we will do it.” That applied to going smoke free in every workplace and opening syringe exchange programmes in communities that were reluctant to have them. That applied to running hard-hitting anti-tobacco advertisements and banning artificial trans-fatty acids from food served in restaurants. However, political leadership alone will not achieve results. You also need technical rigour and operational excellence. With these three things, you can accomplish great things in public health.

Q: What are the barriers to implementing programmes that can save lives?

A: Lack of good surveillance is an important one. When I was running the tuberculosis control programme in NYC, we had terrible budget cuts and I remember wondering, if I had to cut many things, what would I keep? The last thing you close is surveillance. That means getting data in from the front lines, sifting, analysing and using it to improve programme performance. 

Q: For example? 

A: When I became health commissioner, I was shocked to find that NYC had no surveillance system for noncommunicable diseases (NCDs) other than vital registration, which was not sufficiently accurate with regard to cause of death. We launched a community health survey to generate these data. The first year we added questions on West Nile virus, which had emerged in NYC. After looking at the data, we realized that we’d spent a lot on public education that was ineffective, so I stopped that programme, confident no one would get sick. We ran our information systems in real time for two reasons: if you are successful, you can defend your programme; if not, you can improve it or replace it with something better.

Q: At the US Centers for Disease Control and Prevention (CDC), how did you protect people’s health in spite of the powerful food and soft drinks industries?

A: The struggle for health is not over, lots more needs to be done. Many of the policies that I wished had happened on food and drink did not happen because of industry pressure. Politics and public health are the art of the possible: you figure out what can be done and how, and then do it. I tried to identify winnable battles – areas with a high health burden that were amenable to interventions, but where additional, focused effort could achieve results in the short term. CDC identified six of these – tobacco control, improved nutrition, reducing hospital-associated infections, preventing HIV, reducing teen pregnancy, and reducing motor vehicle crash deaths. Every organization has limited resources, so you need to focus on areas where you can make the biggest difference. We achieved our goals in 70% of these battles. We made progress, though not as much as I’d have liked.

Q: How can economies and commercial sectors be re-oriented towards health?

A: Health-care costs account for a large and increasing proportion of societal spending, and unless this spending is made more efficient and prioritizes maximizing health, economies don’t grow as quickly as they could. Business leaders can understand that they have a vested interest in a healthy community and a healthy workforce, and an important role to play in this.

Q: Drug overdoses are the leading cause of death for US citizens aged under 50 years. What did you do at CDC to stop the epidemic of opioid dependency?

A: In medical school we were taught that if you give an opioid to a patient in pain, he or she would not become addicted. This is wrong. A couple of doses can lead to addition and a couple of pills too many, can kill you. To make matters worse, we now have a huge amount of illicit fentanyl and heroin coming into the country. Like many problems, it’s easier to prevent it happening than to control it once it has emerged. At CDC, I first declared there was an epidemic of prescription opioid overdose and death in 2011. That was a shock to people. In response, we established good information systems to show who is using opioids, how much, who is using too much and which doctors are prescribing too much, so that states could take action.

Q: In the media people often talk about the need for individuals to make the right lifestyle choices to improve their health. Is this useful when trying to reduce NCDs?

A: Not entirely. Wouldn’t the world be great if, by adulthood, people are not addicted to tobacco, alcohol or drugs; haven’t experienced mental or physical trauma; are a healthy weight; eat healthily and are physically active; haven’t been exposed to toxins in their environment; and have had the right amount of sodium so that food tastes good without increasing blood pressure? On the other hand, you can’t absolve the individual. Sometimes in public health we do that. We should shape our environment so that if people go with the flow, they will be more likely to have a healthy lifestyle. We also have to make the incentives right. If cigarettes are expensive, hard to buy and you can’t smoke in public places, kids are less likely to start smoking. It’s our collective responsibility, as society, to ensure that the default value is the healthy value while encouraging individuals to act in favour of their own health.

“NCDs are often neglected because they don’t seem urgent … we need to show success.”

Q: Many low- and middle-income countries are facing a double burden of infectious diseases and NCDs. How can they re-orient their health services towards addressing both problems?

A: In public health we were asleep at the switch from communicable to NCDs and our progress fighting NCDs is somewhere between stalled and slow. NCDs now kill far more people worldwide than communicable diseases. At Resolve to Save Lives, we focus on three aspects of cardiovascular health: getting hypertension under control, eliminating artificial trans fat from the food-chain, and sodium reduction. Each is selected for maximum impact. We want to increase hypertension control globally from the current 14% of people with their blood pressure under control to 50%; to reduce sodium consumption by 30% and to eliminate artificial trans fat from the food-chain; doing these three things will save 100 million lives over 30 years.

Q: Why those three things?

A: High blood pressure is one of the world’s leading killers, and the medications to control it are inexpensive, safe and effective. If countries get this right, the impact will be far greater than anything else they do for adults at the primary care level. Sodium reduction takes a different course in different countries. For example, China’s Shangdong province as well as Finland and the United Kingdom [of Great Britain and Northern Ireland] have all made progress. More countries need to try things, study them, document successes and scale them up. Artificial trans fat is a toxic chemical added to your food without your knowledge and accounts for an estimated 540 000 deaths globally each year. Denmark and NYC have shown that you can eliminate trans fat from the food supply without changing taste or reducing food variety. You can say there are more important issues in responding to NCD epidemics in countries, but – after tobacco control – none are more amenable to intervention than these three.

Q: Can you tell us about your work at Resolve to Save Lives with WHO on NCDs?

A: We are providing investment and technical support to several countries to help them scale up proven strategies. We support the cardiovascular components of the WHO Global Hearts Initiative to improve cardiovascular health and the Shake technical package for salt reduction. We are also helping these countries implement effective evaluation and surveillance systems. NCDs are often neglected because they don’t seem urgent, and the issue seems hopeless to some policy-makers. We need to show success. If we can help double or triple the hypertension control rate in the next few years, if we can reduce sodium and eliminate artificial trans fat – we will be helping WHO to improve health and save millions of lives.

